# Selinexor in combination with carboplatin and paclitaxel in patients with advanced solid tumors: Results of a single-center, multi-arm phase Ib study

**DOI:** 10.1007/s10637-021-01188-1

**Published:** 2021-09-25

**Authors:** Kyaw Z. Thein, Daniel D. Karp, Apostolia Tsimberidou, Jing Gong, Selma Sulovic, Jatin Shah, Denái R. Milton, David S. Hong, Filip Janku, Lacey McQuinn, Bettzy A. Stephen, Rivka Colen, Brett W. Carter, Timothy A. Yap, Sarina A. Piha-Paul, Siqing Fu, Funda Meric-Bernstam, Aung Naing

**Affiliations:** 1 Department of Investigational Cancer Therapeutics, The University of Texas MD Anderson Cancer Center, Houston, TX USA; 2 Division of Hematology and Medical Oncology, Oregon Health and Science University/ Knight Cancer Institute, Portland, OR USA; 3 Karyopharm Therapeutics, Newton, MA USA; 4 Department of Biostatistics, The University of Texas MD Anderson Cancer Center, Houston, TX USA; 5 Department of Diagnostic Radiology, The University of Texas MD Anderson Cancer Center, Houston, TX USA; 6 Department of Thoracic Imaging, Division of Diagnostic Imaging, The University of Texas MD Anderson Cancer Center, Houston, TX USA

**Keywords:** Selinexor, Carboplatin, Paclitaxel, Metastatic solid tumors, Selective inhibitor of nuclear export (SINE)

## Abstract

*Background.* Carboplatin and paclitaxel (CT) is one of the standard chemotherapy regimens used in various tumor types. Preclinical models have suggested that selinexor, a first-in-class oral potent selective inhibitor of nuclear export Exportin-1, and CT exerts antitumor activity in multiple malignancies. *Methods.* This was a single-center, multi-arm phase Ib study utilizing a “basket type” expansion. CT and selinexor was employed as one of the 13 parallel arms. Advanced relapsed/refractory solid tumors following standard therapy or where the addition of selinexor to standard regimens deemed appropriate, were eligible. *Results.* Of 13 patients treated, 12 patients were evaluable for response. The most common cancers were breast *(n* = *4),* esophageal *(n* = *2),* ovarian *(n* = *2)* and non-small cell lung cancers *(n* = *2).* All 13 patients had at least one treatment-related adverse events (TRAEs) and the most common were neutropenia (85%), leukopenia (85%), thrombocytopenia (85%), anemia (69%), nausea (54%), vomiting (46%), and fatigue (46%). One patient at 60 mg QW experienced DLT with grade 3 nausea and vomiting lasting 3 days. Unconfirmed partial response (uPR) was observed in 3 patients; one patient each with esophageal, breast, and ovarian cancer. One patient with esophageal adenocarcinoma had confirmed PR, however, was discontinued from the study due to clinical progression. Five patients achieved stable disease (SD). Disease control rate was 8%. Majority of patients (77%), including two patients who had uPR, had prior exposure to carboplatin and/or paclitaxel. Time-to-treatment failure (TTF) ranged from 1 to 153 weeks. *Conclusion.* The RP2D of selinexor was 60 mg QW in combination with CT. The combination conferred viable clinical activity with durable objective responses which should further be explored in tumor types for which CT is used as standard of care. *Trial information.* ClinicalTrials.gov Identifier: NCT02419495. Sponsor(s): Karyopharm Therapeutics. (Trial registration: NCT02419495. Registered 14 April 2015, https://clinicaltrials.gov/ct2/show/NCT02419495).

## Introduction

Nuclear export proteins, known as exportins, regulate the conveyance of bulky cargo molecules through the nuclear pore. This is crucial in preserving cellular homeostasis and is paramount in developing as a therapeutic target in cancer drug development [1]. There are at least seven nuclear export proteins and Exportin-1 (XPO1), also called chromosome region maintenance 1 (CRM1), is the most highly characterized exportin. XPO1 mediates the translocation of more than 200 regulatory proteins, such as many tumor suppressor proteins (TSP) from the nucleus into the cytoplasmic compartment of the cell [1–3]. The derangement in the transport mechanism with upregulating in the level of XPO1 was implicated in tumorigenesis in various malignancies, at least in part by functionally inactivating TSPs by removing them from the nucleus [4, 5]. Moreover, overexpression of XPO1 was shown to contribute to poor outcome in many tumors including both hematologic and solid cancers [4–7]. Selective inhibitors of nuclear export (SINE) were developed to modulate this synchrony by selectively blocking XPO1, resulting in intranuclear accumulation and functional activation of TSP. This restores cell cycle checkpoints and halts tumor growth, leading to the selective apoptosis of cancer cells [8–12].

Selinexor (KPT-330) is a first-in-class, orally bioavailable, potent selective inhibitor of nuclear export (SINE) compound which forms a reversible covalent bond with cysteine 528 residue of the XPO1 cargo-binding pocket, is approved by FDA in multiple myeloma and diffuse large B cell lymphoma [13, 14]. While DNA damaging agents transiently activate multiple TPS, high levels of XPO1 in tumors lead to rapid nuclear export of these proteins, extinguishing their tumor suppressor function. Selinexor was shown to significantly inhibit tumor growth in preclinical models by selectively blocking XPO1-mediated nuclear export leading to nuclear retention and functional activation of TSP and hindering DNA damage repair (DDR) mechanisms [7, 15]. Furthermore, selinexor was shown to provide synergistic activity when combined with DNA damaging therapeutics in solid tumors [15–18].

To further investigate the safety, tolerability and clinical activity of selinexor in combination with standard therapies, we conducted an open label, single-center, multi-arm phase Ib of selinexor in combination with standard chemotherapy in patients with advanced or metastatic solid tumors. Herein, we are reporting selinexor in combination with carboplatin and paclitaxel (CT) in patients with advanced or metastatic solid tumors.

### Patients

Adult (age ≥ 18 years) patients with histologically documented, advanced or metastatic solid tumors (excluding brain tumors) whose tumors were unresponsive or had relapsed following prior systemic therapy or where the addition of selinexor to standard chemotherapy deemed appropriate and acceptable were eligible. Other key inclusion criteria included Eastern Cooperative Oncology Group (ECOG) performance status of 0 or 1 and adequate organ function. The number of prior treatments was not limited. Patients in the study had to have at least one measurable target lesion as defined by Response Evaluation Criteria in Solid Tumors (RECIST v1.1) [19, 20] criteria for solid tumors, except for patients with castrate resistant prostate cancer (CRPC) where prostate cancer working group 2 (PCWG2) criteria was utilized [21]. Key exclusions were patients with primary CNS tumor or active CNS tumor involvement, evidence of complete or partial bowel obstruction or needing total parenteral nutrition, prior treatment with an agent targeting the exportin, and unstable cardiovascular functions.

The primary objective was to establish the safety and tolerability of selinexor when given in combination with standard chemotherapy regimens; secondary objectives included determining the disease control rate (DCR) and progression free survival (PFS) of selinexor administered with standard chemotherapy treatments. The primary efficacy parameter was the tolerability according to National Cancer Institute Common Terminology Criteria for Adverse Events (CTCAE) version 4.03 and the secondary parameters were clinical benefit rate (CBR; percentage of complete response [CR], partial response [PR] plus stable disease [SD]), DCR (percentage of CR, PR plus SD for at least 6 months, assessed according to RECIST 1.1 criteria), the objective tumor response rate (CR + PR), assessed according to RECIST 1.1 criteria and PFS defined as the time between the cycle 1 start date and the date of disease progression or death, whichever is reported first.

### Study design and treatment

This was an open-label, single-center, multi-arm phase Ib of selinexor in combination with standard chemotherapy or immunotherapy treatments to determine the dose-limiting toxicities (DLTs) and maximum tolerated dose (MTD) of selinexor and further explore the safety and tolerability of the MTD in patients with advanced or metastatic solid tumors (ClinicalTrial.gov identifier: NCT02419495). The study was conducted in multi-arms utilizing a standard 3 plus 3 design and a “basket type” expansion. Selinexor in combination with carboplatin and paclitaxel was employed as one of the 13 parallel arms. While carboplatin was dosed at AUC4 along with paclitaxel at 175 mg/m^2^ intravenously every 3 weeks, selinexor was dosed at 60 mg twice weekly (BIW) orally on days 1, 3, 8, and 10 of each 21-day cycle as well as 40–60 mg once weekly (QW) on days 1, 8, and 15. The study protocol was approved by the Institutional Review Board or Independent Ethics Committee at the MD Anderson Cancer Center and was conducted in accordance with the Declaration of Helsinki, Good Clinical Practice, and all local and federal regulatory guidelines. All patients signed informed consent prior to enrolling onto the study.

### Study assessments

Tumor response was assessed using RECIST v1.1. Baseline imaging was done within 30 days of treatment initiation. Repeat imaging (using the same methodology as at baseline) was obtained every 9 weeks. Treatment-emergent adverse events (TEAEs) and treatment-related adverse events (TRAEs) were graded using the Common Terminology Criteria for Adverse Events (CTCAE) version 4.03. DLT was defined as any selinexor-related grade 4 hematologic adverse event, grade ≥ 3 thrombocytopenia associated with clinically significant bleeding, febrile neutropenia or non-hematologic adverse event grade ≥ 3 in severity per CTCAE (v 4.03) despite optimal supportive medications, excluding electrolyte abnormalities that are reversible, asymptomatic or hair loss which is not dose-limiting. The MTD was defined as the highest dose level at which ≤ 33% of patients experience DLTs during cycle 1. After the MTD was defined in each schedule, the study was extended to include additional evaluable patients at the MTD. A safety monitoring committee comprised of investigators and the study sponsor reviewed all safety information and made consensus decisions about dose escalation.

### Statistical methods

Patient characteristics, TEAEs, TRAEs, tumor response, and time-to-treatment failure (TTF) were summarized using descriptive statistics. PFS time was computed from cycle 1 start date to the date of disease progression or death (if the patient died without disease progression), or the last evaluation date. Patients who were alive and did not experience progression of disease at the last follow-up date were censored. Overall survival time (OS) was computed from cycle 1 start date to the last-known vital sign. Patients alive at the last follow-up date were censored. The Kaplan–Meier method was used to estimate PFS and OS. All statistical analyses were performed using SAS 9.4 for Windows (Copyright © 2002–2012 by SAS Institute Inc., Cary, NC).

## Results

### Patient characteristics

A total of 13 patients with advanced, metastatic malignancies were enrolled between June 2015 and October 2017. Demographic and clinical characteristics of all patients enrolled are summarized in Table 1. The median age of patients was 57 (range, 41–71 years), with 61.5% female and 38.5% male. The median number of prior systemic therapies was 3 (range, 0–8). The most common types of cancer enrolled were breast *(n* = *4),* esophageal *(n* = *2),* ovarian *(n* = *2)* and non-small cell lung cancer *(n* = *2).* One patient was dosed at BIW dosing of selinexor while six patients received dose at 60 mg QW, and six patients were dosed at 40 mg QW. The median number of cycles completed for all patients was 4 (range, 0–43). For patients with SD or better, the median number of cycles completed was 5 (range, 2–43).

### Safety and tolerability

Twelve patients are no longer on the study whereas a patient with metastatic lung adenocarcinoma continued the study until the data cut off in May 2020. Progression of disease accounted for the majority of patient withdrawals from the study and clinically unacceptable TEAEs contributed to withdrawal of one patient. All 13 patients had at least one TEAE. Summaries of TEAEs and TEAEs related to selinexor are presented in Table 2 and Table 3**.** The most common TEAEs were anemia (85%), neutropenia (85%), leukopenia (85%), thrombocytopenia (85%), fatigue (62%), nausea (54%), hypomagnesemia (54%), and peripheral motor or sensory neuropathy (54%). The most prevalent grade ≥ 3 TEAEs were neutropenia (69%), thrombocytopenia (54%), leukopenia (46%), and anemia (15%). TEAEs related to selinexor were reported in all patients (100%). The most common TRAEs were neutropenia (85%), leukopenia (85%), thrombocytopenia (85%), anemia (69%), nausea (54%), vomiting (46%), fatigue (46%), hyponatremia (31%), and peripheral motor or sensory neuropathy (31%). The most common grade 3/4; deemed to be related to selinexor, adverse events were hematological laboratory abnormalities: neutropenia (69%), thrombocytopenia (54%), leukopenia (46%), and anemia (15%). Table 4 depicted the detailed TEAEs related to selinexor at each dose level. Hematological laboratory abnormalities were common when selinexor was dosed 60 mg QW and above. One patient at 60 mg QW experienced DLT with grade 3 nausea and vomiting lasting 3 days. Four patients reported having serious adverse events (SAE). Two patients had at least one SAE considered related to selinexor treatment; one patient had grade 3 dehydration and hyponatremia while another patient experienced grade 3 nausea and vomiting with adult failure to thrive. The SAEs experienced by the other two patients were considered unrelated to selinexor treatment; one patient had grade 3 lung infection and co-occurring grade 2 pneumonitis and one patient experienced grade 3 infections and infestations (other). No patients died during the course of the study.

### Antitumor activity

**Fig. 1:**
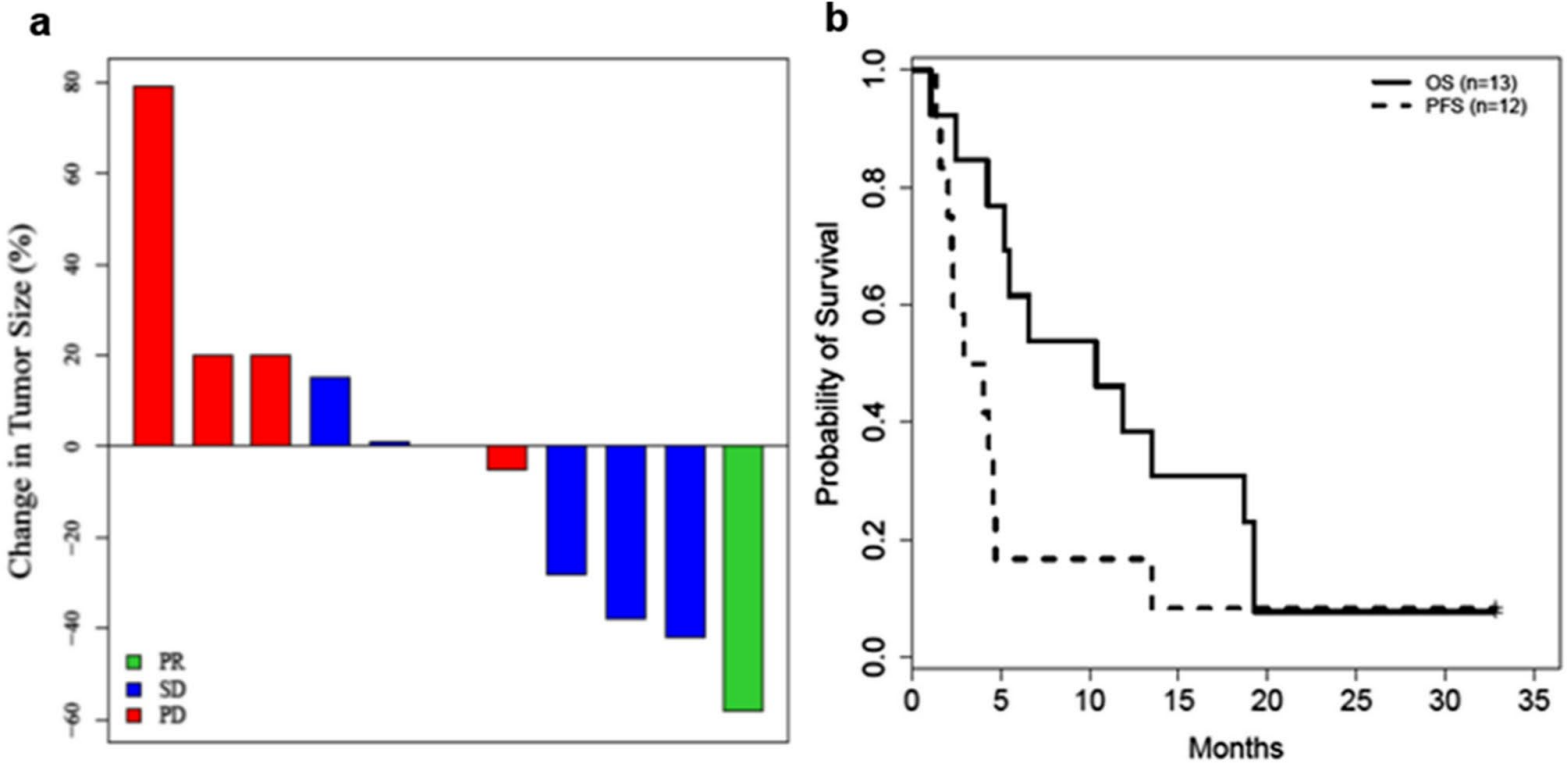
**a** Waterfall plot of maximum change in tumor measurements (per RECIST v1.1) for evaluable patients. **b** Kaplan-Meier plot showing progression free survival (PFS) for evaluable patients and overall survival (OS) for all treated patients

Best overall tumor response is shown in Fig. 1a**.** Thirteen patients enrolled in the study had measurable disease, but one patient had not completed their first restaging scans due to earlier withdrawal of consent. Twelve patients completed their first restaging scans per protocol and considered as efficacy evaluable patients. Unconfirmed PR was noted in three patients; one patient each with hormone receptor-positive, HER-2 negative breast, ovarian, and squamous cell esophageal cancer. Although one confirmed PR was observed in a patient with adenocarcinoma of esophagus with -58% in tumor reduction, patient was discontinued from the study due to clinical progression. Reduction in tumor ranged from -38% to -58% for the four patients experiencing PR as best response. Five patients (42%) achieved SD contributing to CBR of 42%. Of the five SD patients, one patient with metastatic lung adenocarcinoma who had progressed on prior three lines of therapies including carboplatin and pemetrexed, nivolumab followed by ramucirumab plus paclitaxel, had exceptionally durable stable disease (currently ongoing at the data cut off with TTF of 35.4 months and had completed total of 43 cycles of treatments) despite the disease being categorized as non-measurable. Hence, the DCR was 8%. Majority of patients (77%), including two patients who had uPR, had prior exposure to carboplatin and/or paclitaxel. TTF for patients who achieved durable disease control ranged from 10 to 153 weeks. Overall, the median TTF was 13 weeks (range, 1 to 153 weeks). For patients with SD or better, the median TTF was 18 weeks (range, 7 to 153). The median PFS was 3.0 months (95% confidence interval [CI]: 1.6 – 4.5 months) for all patients, while the median OS was 11.9 months (95% CI: 4.2 – 19.2 months) (Fig. 1b).

## Discussion

Most bulky cargo proteins > 40kD including TSPs, and many RNAs require certain transporters to exit through the nuclear pore complex; these carriers are called exportins 1–7 [4]. Emerging data has suggested that tumor cells overexpress Exportin-1 (XPO1), which is the major nuclear export protein in the cell mediating the efflux of tumor suppressor proteins and the methyl-guanine capped mRNA binding protein eIF4E [22, 23]. Active nuclear export of TSPs and eIF4E-bound oncoprotein mRNAs was effectively blocked by SINE compounds which selectively inhibit XPO1 function, leading to nuclear retention of TSP and eIF4E-bound mRNAs, impeding the cancer growth and prompting selective apoptosis of cancer cells [8–12]. Selinexor (KPT-330) is a first-in-class, orally bioavailable, slowly reversible, potent SINE which covalently binds the cysteine 528 residue of the cargo-binding pocket in XPO1 [13, 14]. DNA damage by cytotoxic chemotherapies transiently activate multiple TSPs, high levels of XPO1 in tumors lead to rapid nuclear export of these proteins, extinguishing their tumor suppressor function. In preclinical and earlier clinical studies, selinexor was shown to provide synergistic or additive activity when combined with DNA damaging agents in solid tumors by attenuating DNA damage repair (DDR) mechanism and maintaining nuclear localization and functional activation of TSPs [15].

This is the first study reporting selinexor in combination with carboplatin and paclitaxel (CT), which is one of the most commonly used regimens in solid tumors, from the open label, single-center, multi-arm, “basket type” expansion phase IB study. Previously, single agent selinexor had been studied in various solid tumors and has shown either some or limited efficacy [24–27]. Compared to previous single agent selinexor studies where fatigue and hematological laboratory abnormalities were the most common high-grade TRAE ranging from 6 to 21%, greater incidence of high-grade hematological laboratory abnormalities were observed with this combination strategy despite employing the lower dose of standard of care dose of carboplatin (AUC of 4 or 5) and paclitaxel (175 mg/m^2^ every 3 weeks). Majority of patients (92%) received once weekly selinexor dosing regimen in this study in contrast to prior single agent selinexor studies where twice weekly dosing regimens were implemented. In addition, detailed TEAEs related to selinexor at each dose level in our study characterized toxicity as a function of dose level when selinexor combined with carboplatin and paclitaxel. Hematological laboratory abnormalities and gastrointestinal toxicities were notable when selinexor was dosed 60 mg QW and above. In recent phase II KING study where selinexor monotherapy was given in a cohort of patients with recurrent glioblastoma, once weekly selinexor was better tolerated with antiemesis prophylaxis contributing less high-grade toxicities while attaining some antitumor activity with disease control [26]. Proper utility of growth factors and optimizing supportive care is crucial in this combination strategy. Overall, all 13 patients had at least one TEAE. The most common TEAEs were hematological laboratory abnormalities, fatigue, nausea, hypomagnesemia, and peripheral motor or sensory neuropathy where the latter was contributed from chemotherapy. The most prevalent grade ≥ 3 TEAEs were hematological laboratory abnormalities. TEAEs related to selinexor were neutropenia (85%), leukopenia (85%), thrombocytopenia (85%), anemia (69%), nausea (54%), vomiting (46%), and fatigue (46%). The most common grade ≥ 3 adverse events; deemed to be related to selinexor were the following hematological laboratory abnormalities: neutropenia (69%), thrombocytopenia (54%), and leukopenia (46%). In our study, one patient at 60 mg once weekly experienced DLT with grade 3 nausea and vomiting lasting 3 days. Four patients reported having SAEs. Two patients had at least one SAE considered related to selinexor treatment; one patient had grade 3 dehydration and hyponatremia while another patient experienced grade 3 nausea and vomiting with adult failure to thrive. The SAEs experienced by the other two patients were considered unrelated to selinexor treatment; one patient had grade 3 lung infection and co-occurring grade 2 pneumonitis and one patient experienced grade 3 infections and infestations.

In the initial first-in-human study in patients with advanced refractory solid tumors, single agent selinexor induced a 4% CR + PR rate with 17% SD ≥ 4 months; responses were observed in melanoma, colorectal cancer, ovarian cancer, prostate cancer, thymoma, and cervical cancer [28]. In patients with metastatic triple-negative breast cancer, the study was terminated early due to lack of objective responses despite 3 of 10 patients (30%) had SD for ≥ 12 weeks [24]. In another phase Ib study of selinexor in patients with refractory soft tissue or bone sarcoma, 30 of 52 patients (58%) achieved SD while 17 (33%) patients had durable response lasting more than 4 months and the activity is particularly noted in dedifferentiated liposarcoma [26]. In a phase II study employing selinexor in heavily pretreated gynecological cancers, disease control rate (CR + PR + SD ≥ 12 weeks) was reported as 30%, 35% and 24% in ovarian, endometrial and cervical cancers, respectively, with PR + CR in 8% in ovarian cancer, 9% in endometrial cancer and 4% in cervical cancer [25].

In our study, no patient experienced CR. Unconfirmed PR was observed in three patients: one patient each with hormone receptor-positive, HER-2 negative breast, ovarian, and squamous cell esophageal cancer. A patient with adenocarcinoma of esophagus had confirmed PR with -58% in tumor reduction, however this patient was reported to have clinical progression and was discontinued from the study. Reduction in tumor ranged from -38% to -58% for the four patients experiencing PR as best response. Five patients (42%) achieved SD contributing to CBR of 42%. Among five patients who achieved SD, one patient with metastatic lung adenocarcinoma who had progressed on prior three lines of therapies including carboplatin + pemetrexed, nivolumab followed by ramucirumab + paclitaxel, had exceptionally durable stable disease (currently ongoing at the data cut off with TTF of 35.4 months and had completed total of 43 cycles of treatments) despite the disease being categorized as non-measurable. Hence, the DCR was 8%. The histology of the two patients with esophageal cancer included squamous cell and adenocarcinoma; neither patient had any prior exposure with carboplatin and/or paclitaxel. However, the other two patients with uPR (hormone receptor-positive, HER-2 negative breast cancer and ovarian cancer), did have prior exposure with carboplatin and/or paclitaxel. There were two patients with ovarian cancer in the cohort and both patients including the one who achieved uPR, had received prior poly ADP-ribose polymerase (PARP) inhibitor.

## Conclusion

One weekly selinexor can be safely combined with standard carboplatin/paclitaxel and the RP2D of selinexor was 60 mg QW in combination with CT. The combination conferred viable clinical activity with durable objective responses in this heavily pretreated patient population which should further be explored in tumor types for which CT is used as standard of care. These results support the evaluation of the combination of selinexor and CT in patients with carboplatin and paclitaxel naïve disease. Proper utility of growth factors and optimizing supportive care is crucial in this combination strategy.

**Table 1:** Patients baseline demographics and disease characteristics

	**Carboplatin 5AUC and ** **Paclitaxel 175mg/m**^**2**^** IV Q3W**			**Carboplatin 4AUC and Paclitaxel 175mg/m**^**2**^** IV Q3W**		
**Characteristic**	**Selinexor 60mg PO BIW *(n=1)***	**Selinexor 60mg PO QW *(n=4)***	**Selinexor 40mg PO QW *(n=1)***	**Selinexor 40mg PO QW *(n=5)***	**Selinexor 60mg PO QW *(n=2)***	**All Patients** ***(N= 13)***
**Age at consent (years)**						
Median Range	57	60.6 (41.8-70.6)	45	58 (50-71)	55.9 (50.4-61.5)	57.6 (41.8 - 71.6)
**Gender, *n (%)***						
Male	0	1 (25)	0	3 (60)	1 (50)	5 (38)
Female	1 (100)	3 (75)	1 (100)	2 (40)	1 (50)	8 (62)
**Race/ethnicity, *n (%)***						
White	0	4 (100)	1 (100)	1 (20)	2 (100)	8 (62)
Hispanic	1 (100)	0	0	2 (40)	0	3 (23)
Black	0	0	0	2 (40)	0	2 (15)
Asian	0	0	0	0	0	0
**ECOG performance status, *n (%)***						
0	1 (100)	0	0	0	0	1 (8)
1	0	4 (100)	1 (100)	5 (100)	2 (100)	12 (92)
**Primary tumor, *n (%)***						
Ovarian	1 (100)	1 (25)	0	0	0	2 (15)
Breast	0	1 (25)	0	2 (40)	1 (50)	4 (31)
Colorectal Cancer	0	0	0	0	0	0
Endometrial/fallopian	0	1 (25)	0	0	0	1 (8)
Lung	0	1 (25)	0	1 (20)	0	2 (15)
Neuroendocrine	0	0	0	0	0	0
Pancreas	0	0	0	1 (20)	0	1 (8)
Esophageal	0	0	0	1 (20)	1 (50)	2 (15)
Head & Neck/salivary gland	0	0	0	0	0	0
Liver/ cholangiocarcinoma	0	0	1 (100)	0	0	1 (8)
Sarcoma	0	0	0	0	0	0
Prostate	0	0	0	0	0	0
Others	0	0	0	0	0	0
**Prior lines of systemic therapies, *n (%)***						
0 - 1	0	0	0	1 (20)	0	1 (8)
2 - 3	0	2 (50)	1 (100)	2 (40)	2 (100)	7 (54)
4 - 5	1 (100)	2 (50)	0	0	0	3 (23)
> 5	0	0	0	2 (40)	0	2 (15)
**Prior exposure to carboplatin and/or paclitaxel, *n (%)***						
Yes	1 (100)	4 (100)	0	4 (80)	1 (50)	10 (77)
No	0	0	1 (100)	1 (20)	1 (50)	3 (23)

*AUC* area under the curve, *BIW* twice weekly dosing schedule, *ECOG* Eastern Cooperative Oncology Group, *IV* intravenous, *PO* oral, *Q3W* 3 weekly dosing schedule, *QW* weekly dosing schedule

**Table 2:** Summary of treatment emergent adverse events in the phase I safety population

**Measure, *n (%)***	**All Patients** ***(N= 13)***
≥ 1 TEAE	13 (100)
≥ 1 TRAE (selinexor)	13 (100)
Grade 3/4 TEAE	12 (92)
Grade 3/4 TRAE (selinexor)	11 (85)
SAE*	4 (31)
≥ 1 TRSAE (selinexor)*	2 (23)
≥ 1 DLT**	1 (8)
Discontinued due to ≥ 1 TEAE	1 (8)

*DLT*, dose limiting toxicity, *SAE* serious adverse events, *TEAE* treatment-emergent adverse events, *TRAE* treatment-related adverse events, *TRSAE* treatment-related serious adverse events

*Four patients were reported to have SAEs; two had at least one TRSAE due to selinexor treatment; one patient had grade 3 dehydration and hyponatremia and one patient experienced grade 3 nausea and vomiting with adult failure to thrive. One patient experienced grade 3 infections and infestations (other) and one patient experienced grade 3 lung infection and co-occurring grade 2 pneumonitis which were considered unrelated to Selinexor treatment

**One patient receiving Selinexor 60mg once weekly experienced dose limiting grade 3 nausea and vomiting lasting 3 days

**Table 3:** Summary of treatment-emergent and -related adverse events in all grades of severity

***N (%)***	**Treatment emergent adverse events (TEAE)**		**Selinexor Treatment related adverse events (TRAE)**	
	**All grades**	**Grade 3/4**	**All grades**	**Grade 3/4**
Anemia	11 (85)	2 (15)	9 (69)	2 (15)
Leukopenia	11 (85)	6 (46)	11 (85)	6 (46)
Neutropenia	11 (85)	9 (69)	11 (85)	9 (69)
Thrombocytopenia	11 (85)	7 (54)	11 (85)	7 (54)
Constipation	6 (46)	0	2 (15)	0
Diarrhea	4 (31)	0	2 (15)	0
Nausea	7 (54)	1 (8)	7 (54)	1 (8)
Vomiting	6 (46)	1 (8)	6 (46)	1 (8)
Elevated AST/ALT	6 (46)	1 (8)	3 (23)	0
Fever	4 (31)	0	0	0
Fatigue	8 (62)	0	6 (46)	0
Anorexia	4 (31)	0	3 (23)	0
Hyponatremia	4 (31)	1 (8)	4 (31)	1 (8)
Hypomagnesemia	7 (54)	0	2 (15)	0
Peripheral motor/ sensory neuropathy	7 (54)	0	4 (31)	0
Dyspnea	3 (23)	1 (8)	1 (8)	0
Cough	5 (38)	0	0	0
Elevated CPK	2 (15)	1 (8)	1 (8)	1 (8)
Arthralgia	1 (8)	0	1 (8)	0
Infection or infestation	5 (38)	4 (31)	0	0

*ALT* alanine aminotransferase, *AST* aspartate aminotransferase, *CPK* creatine phosphokinase

**Table 4:** Summary of treatment-related adverse events in each dose level

***N (%)***	**Selinexor Treatment related adverse events (TRAE)**									
	**S 40mg QW, ** **C 4 AUC, ** **P 175 mg/m**^**2**^ **(*N = 5*) **		**S 40mg QW, ** **C 5 AUC, ** **P 175 mg/m**^**2**^ **(*N = 1*) **		**S 60mg QW, ** **C 4 AUC, ** **P 175 mg/m**^**2**^ **(*N = 2*) **		**S 60mg BIW, ** **C 5 AUC, ** **P 175 mg/m**^**2**^ **(*N = 1*)**		**S 60mg QW, ** **C 5 AUC, ** **P 175 mg/m**^**2**^ **(*N = 4*)**	
	**Grade 1/2**	**Grade 3/4**	**Grade 1/2**	**Grade 3/4**	**Grade 1/2**	**Grade 3/4**	**Grade 1/2**	**Grade 3/4**	**Grade 1/2**	**Grade 3/4**
Anemia	1	1	0	0	1	1	1	0	4	0
Leukopenia	1	2	1	0	1	1	1	0	1	3
Neutropenia	1	2	0	1	1	1	0	1	0	4
Thrombocytopenia	2	1	1	0	0	2	1	0	0	4
Constipation	1	0	0	0	0	0	1	0	0	0
Diarrhea	2	0	0	0	0	0	0	0	0	0
Nausea	2	0	0	0	0	0	1	0	3	1
Vomiting	2	0	0	0	0	0	1	0	2	1
Elevated AST/ALT	1	0	0	0	1	0	0	0	1	0
Fever	0	0	0	0	0	0	0	0	0	0
Fatigue	2	0	1	0	0	0	0	0	3	0
Anorexia	0	0	0	0	0	0	1	0	2	0
Hyponatremia	1	0	0	1	0	0	1	0	1	0
Hypomagnesemia	0	0	0	0	0	0	0	0	2	0
Peripheral motor/ sensory neuropathy	2	0	0	0	1	0	0	0	1	0
Dyspnea	1	0	0	0	0	0	0	0	0	0
Cough	0	0	0	0	0	0	0	0	0	0
Elevated CPK	0	0	0	0	0	0	0	1	0	0
Arthralgia	1	0	0	0	0	0	0	0	0	0
Infection or infestation	0	0	0	0	0	0	0	0	0	0

## Data Availability

-The datasets used and/or analyzed during the current study are available from the corresponding author on reasonable request.

## Abbreviations

CT: Carboplatin + taxol
TRAEs: Treatment-related adverse events
SD: Stable disease
DCR: Disease control rate
CBR: Clinical benefit rate
CR: Complete response
PR: Partial response
uPR: Unconfirmed PR
TTF: Time-to-treatment failure
XPO1: Exportin-1
CRM1: Chromosomal region maintenance 1
TSP: Tumor suppressor proteins
SINE: Selective inhibitors of nuclear exports
DDR: DNA damage repair
ECOG: Eastern cooperative oncology group
RECIST v1.1: Response evaluation criteria in solid tumors version 1.1
CRPC: Castrate resistant prostate cancer
PCWG2: Prostate cancer working group 2
CTCAE: Common terminology criteria for adverse events
PFS: Progression-free survival
DLT: Dose-limiting toxicities
MTD: Maximum tolerated dose
BIW: Twice weekly
QW: Weekly
TEAEs: Treatment-emergent adverse events
OS: Overall survival
SAEs: Serious adverse events
PARP: Poly ADP-ribose polymerase

## Code availability

**-**Not applicable.
