# Correction to: Selinexor in combination with carboplatin and paclitaxel in patients with advanced solid tumors: results of a single‑center, multi‑arm phase Ib study

**DOI:** 10.1007/s10637-021-01194-3

**Published:** 2021-11-03

**Authors:** Kyaw Z. Thein, Daniel D. Karp, Apostolia Tsimberidou, Jing Gong, Selma Sulovic, Jatin Shah, Denái R. Milton, David S. Hong, Filip Janku, Lacey McQuinn, Bettzy A. Stephen, Rivka Colen, Brett W. Carter, Timothy A. Yap, Sarina A. Piha‑Paul, Siqing Fu, Funda Meric‑Bernstam, Aung Naing

**Affiliations:** 1 Department of Investigational Cancer Therapeutics, The University of Texas MD Anderson Cancer Center, Houston, TX USA; 2 Division of Hematology and Medical Oncology, Oregon Health and Science University/ Knight Cancer Institute, Portland, OR USA; 3 Karyopharm Therapeutics, Newton, MA USA; 4 Department of Biostatistics, The University of Texas MD Anderson Cancer Center, Houston, TX USA; 5 Department of Diagnostic Radiology, The University of Texas MD Anderson Cancer Center, Houston, TX USA; 6 Department of Thoracic Imaging, Division of Diagnostic Imaging, The University of Texas MD Anderson Cancer Center, Houston, TX USA

## Correction to: Investigational New Drugs https://doi.org/10.1007/s10637-021–01188-1

The article **Selinexor in combination with carboplatin and paclitaxel in patients with advanced solid tumors: Results of a single‑center, multi‑arm phase Ib study** was originally published electronically on the publisher’s internet portal on 25 September 2021 without open access. The author decided to opt for Open Choice and to make the article an Open Access publication. Therefore, the copyright of the article has been changed to © The Author(s) 2021 and the article is forthwith distributed under a Creative Commons Attribution 4.0 International License (https://creativecommons.org/licenses/by/4.0/), which permits use, sharing, adaptation, distribution and reproduction in any medium or format, as long as you give appropriate credit to the original author(s) and the source, provide a link to the Creative Commons licence, and indicate if changes were made.

The original article has been corrected.

