# Correction to: Selinexor in combination with carboplatin and paclitaxel in patients with advanced solid tumors: results of a single-center, multi-arm phase Ib study

**DOI:** 10.1007/s10637-024-01493-5

**Published:** 2025-02-20

**Authors:** Kyaw Z. Thein, Daniel D. Karp, Apostolia Tsimberidou, Jing Gong, Selma Sulovic, Jatin Shah, Denái R. Milton, David S. Hong, Filip Janku, Lacey McQuinn, Bettzy A. Stephen, Rivka Colen, Brett W. Carter, Timothy A. Yap, Sarina A. Piha-Paul, Siqing Fu, Funda Meric-Bernstam, Aung Naing

**Affiliations:** 1 Department of Investigational Cancer Therapeutics, The University of Texas MD Anderson Cancer Center, Houston, TX USA; 2 Division of Hematology and Medical Oncology, Oregon Health and Science University/ Knight Cancer Institute, Portland, OR USA; 3 Karyopharm Therapeutics, Newton, MA USA; 4 Department of Biostatistics, The University of Texas MD Anderson Cancer Center, Houston, TX USA; 5 Department of Diagnostic Radiology, The University of Texas MD Anderson Cancer Center, Houston, TX USA; 6 Department of Thoracic Imaging, Division of Diagnostic Imaging, The University of Texas MD Anderson Cancer Center, Houston, TX USA

**Correction to: Invest New Drugs**

10.1007/s10637-021-01188-1

In the original published version of the paper, “Selinexor in combination with carboplatin and paclitaxel in patients with advanced solid tumors: Results of a single-center, multi-arm phase Ib study”, 3 patients with unconfirmed partial response (uPR) were considered to have partial response as their best response. However, as PR could not be confirmed in the subsequent scan, best response has now been corrected per RECIST criteria v1.1. for these 3 patients. Two patients are now reported to have stable disease and 1 patient is now reported to have clinical progression of disease as their best response. As a result of these changes, the objective response rate and disease control rate are corrected to be 8% and 17%, respectively. The median progression-free survival (PFS) is 3.5 months (95% confidence interval [CI]: 1.6 – 4.7 months), while the median overall survival (OS) is 10.3 months (95% CI: 4.2 – 18.7 months). To align with protocol-defined endpoints, time to treatment failure (TTF) has been deleted. Figure 1 has been revised accordingly. The authors apologize for the mistake.

**Fig. 1:**
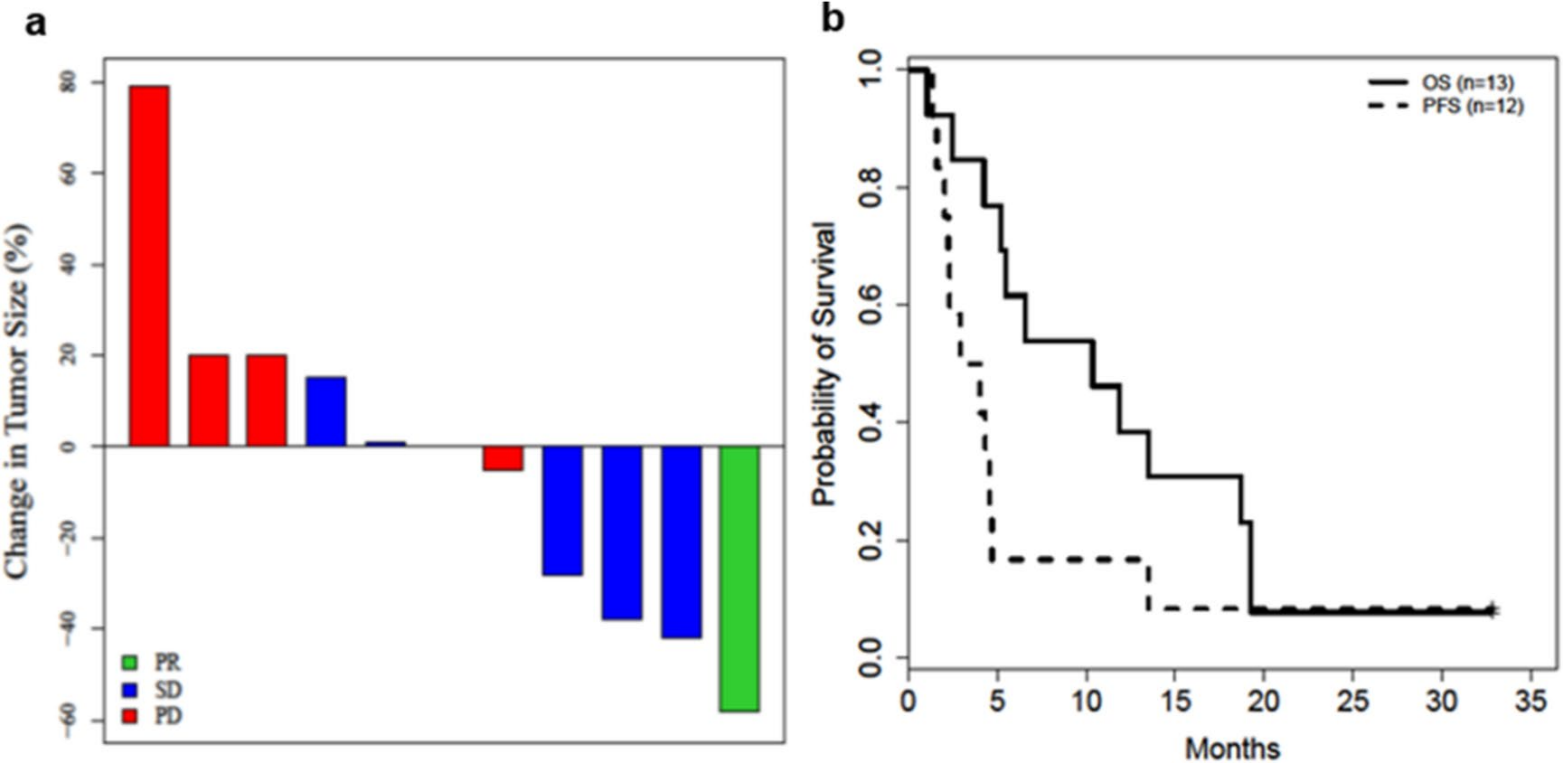
**a** Waterfall plot of maximum change in tumor measurements (per RECIST v1.1) for evaluable patients. **b** Kaplan–Meier plot showing progression-free survival (PFS) for evaluable patients and overall survival (OS) for all treated patients

